# Harnessing neuroimaging-guided transcranial magnetic stimulation for precision therapy in substance use disorders

**DOI:** 10.1038/s41380-025-03024-x

**Published:** 2025-04-16

**Authors:** Smita Sahay, Madhu Vishnu Sankar Reddy Rami Reddy, Charlotte Lennox, Emma Wolinsky, Robert E. McCullumsmith, Tanvir Singh

**Affiliations:** 1 Department of Neurosciences and Psychiatry, University of Toledo College of Medicine and Life Sciences, Toledo, OH 43614 USA; 2 Department of Medicine, University of Toledo College of Medicine and Life Sciences, Toledo, OH 43614 USA; 3 Neuroscience Institute, ProMedica, Toledo, OH 43606 USA

**Keywords:** Neuroscience, Psychology

## Abstract

Substance use disorders (SUDs) are a critical public health challenge characterized by high relapse rates, with existing treatments often proving inadequate. The focus of this review is to provide an update on the current state of transcranial magnetic stimulation (TMS) as a therapeutic intervention for SUDs and discuss neuroimaging-guided TMS practices. This review explores the neurobiology underlying SUDs, emphasizing the roles of the prefrontal cortex, striatal circuits, and dopaminergic pathways, and examines the theory that TMS modulates neurocircuitry to impact addiction-related behaviors. We discuss TMS procedural aspects and provide a comparative analysis of TMS protocols, focusing on repetitive, deep, single-pulse, paired-pulse, and a more recent approach, theta burst stimulation. We review recent randomized clinical trials (RCTs) to demonstrate reductions in cravings and use across SUDs as well as highlight the need for standardized protocols. We emphasize the power of combining neuroimaging techniques to show functional connectivity changes in the brain and identify potential biomarkers predictive of SUD treatment response, an unexplored area of discussion. With these topics, this review highlights the potential of TMS as a versatile and effective therapeutic modality for SUDs, especially when combined with neuroimaging. Key findings emphasize the necessity for future research to address methodological challenges, such as standardizing protocols and optimizing stimulation parameters. The integration of neuroimaging provides insights into functional connectivity changes, enabling enhanced precision and individualized treatment strategies. By validating TMS approaches and incorporating multimodal techniques, this field can advance toward a more robust clinical utility in addressing the complex neurocircuitry of addiction-related behaviors underlying SUDs.

## Introduction

Substance use disorders (SUDs) represent a substantial public health challenge, with recent data revealing an alarming increase in prevalence. In 2020, approximately 40.3 million individuals aged 12 and older in the United States experienced a SUD, a figure that rose to 48.7 million by 2022. Approximately eight million of these individuals had concurrent alcohol and drug use disorders [1, 2]. SUDs frequently coexist with mental health disorders such as anxiety and depression, which affect approximately 20% of patients with SUDs. However, the complications associated with SUDs extend beyond psychological issues, impacting various aspects of life including work and relationships, and leading to severe health problems such as seizures, stroke, respiratory depression, organ failure, cancer, and even death [3].

Current treatment modalities for SUDs encompass detoxification, cognitive and behavioral therapies, and pharmaceutical interventions [3]. Despite these options, several challenges hinder effective treatment, including high relapse rates, high cost of medication and therapy, and an insufficient number of healthcare facilities and healthcare professionals across the United States (U.S.) [4]. In addition, the stigma associated with SUDs and mental health challenges often prevents individuals from seeking professional help, with many attempting to self-manage their conditions. Although stigmatizing attitudes have been slow to change, these retributive viewpoints have been giving way to a cultural and medical consensus that addiction is a treatable chronic disorder of the brain [5, 6].

Transcranial magnetic stimulation (TMS) is a promising non-invasive neuromodulation technique capable of stimulating the brain to produce behavioral effects [7]. TMS has been approved by the U.S. Food and Drug Administration (FDA) for the treatment of major depressive disorder since 2008, obsessive-compulsive disorder since 2017, and most recently, tobacco smoking cessation in the adult population since 2020 [8, 9]. Studies have demonstrated the potential of TMS therapy in reducing cravings, decreasing substance consumption, and mitigating impulsivity, thereby enhancing long-term outcomes for individuals with SUDs [10, 11]. Thus, there has been an exponential growth in the application of TMS to investigate these networks in populations with SUDs including alcohol, cocaine, cannabis, methamphetamine, and opioid use disorders [8].

A multi-dimensional approach to treating SUDs involves a combination of pharmacological, behavioral, psychosocial, and neuromodulation interventions; however, the aim of the present review is to provide an update on TMS as a therapeutic intervention for SUDs. This review provides evidence of TMS interventions targeting specific brain regions and investigates how this approach modulates the underlying neurobiological mechanisms and circuitry implicated in addiction and SUDs. We further analyze and review randomized clinical trials (RCTs) that show efficacy for various protocols such as repetitive, single-pulse, and paired-pulse TMS as wells as theta burst stimulation in SUDs. Finally, and most importantly, we discuss the integration of neuroimaging to uncover functional connectivity changes in the brain and identify potential biomarkers that predict TMS treatment response. This review thoroughly addresses the current state of TMS in SUDs and advocates for standardizing protocols with the use of neuroimaging modalities for the overall advancement of SUD treatment.

## Pathophysiology

At the core of SUDs is the dysregulation of the brain’s dopamine system [12]. Dopamine, a neurotransmitter critical for reward, pleasure, motivation, mood, and cognition, is synthesized by neurons in the substantia nigra and the ventral tegmental area (VTA), which project to various regions of the striatum including the nucleus accumbens (NAC)—a key component of the ventral striatum [13]. The enzymes required for dopamine synthesis are transported from neuronal cell bodies in these midbrain regions to their axon terminals, where the conversion of the amino acid tyrosine to dopamine occurs [13].

The mesocortical pathway, connecting the VTA to the prefrontal cortex (PFC), particularly the dorsolateral prefrontal cortex (DLPFC), regulates stress responses by modulating the production of dopamine [14]. Dysfunction of this pathway leads to the lack of stress resilience [15], a key attribute perpetuating behaviors of addiction. The PFC is an anatomically and functionally heterogeneous brain region that influences cognitive and limbic processing via connections to other subcortical regions and circuits [16]. Thus, key targets for TMS include the DLPFC, crucial for executive function and decision-making, the medial PFC (MPFC), crucial for emotional regulation and social cognition, and the cingulate cortex, which is integral in emotion formation, learning, and memory [17]. In the context of SUDs, these regions are often targeted with the goal of modulating mesocortical and dopaminergic activity to restore cognitive and emotional homeostasis, reduce cravings, and lower the risk of relapse [18–20].

The mesolimbic pathway, connecting the VTA to the NAC, is also central to the brain’s reward system. Upon consumption of substances, this pathway drives a substantial increase in the tonic release of dopamine (i.e., sustained increase in dopamine levels following substance use), which reinforces drug-seeking behavior by providing a powerful reward signal [21]. Heightened dopamine activity leads to neuroadaptations that diminish the baseline (i.e., long-term, resting level) release of dopamine, causing individuals to experience reduced pleasure from normally rewarding activities (i.e., non-drug stimuli) and an increased craving for substances. This phenomenon is often described as a blunted dopamine response at baseline, a hallmark of addiction [22]. TMS has shown promise in modulating this pathway and reducing the heightened reward response that perpetuates pleasure-seeking behavior by targeting the PFC as well as regions connected to the PFC, such as the insular cortex (insula) [23, 24]. The insula, through its role in interoception, influences the mesolimbic pathway and the brain’s reward system by linking sensory cues to rewards and driving addiction-related behaviors [25, 26]. Insula-targeted TMS studies have shown reductions in substance use, cravings, and compulsions in individuals with SUDs [25, 26].

The striatum, particularly the dorsal striatum, is another key region. The nigrostriatal pathway connects the substantia nigra to this region and is primarily involved in motor control. While this pathway is not as directly associated with addiction as are the dopamine modulating mesocortical and mesolimbic pathways, recent work suggests that the non-dopaminergic feedback loops originating from the nigrostriatal pathway contribute to the habit-forming properties of illicit substance use and are central in the rigid behavioral patterns that lead to relapse [27]. The TMS-targeted motor cortex contributes to the physical aspect of habitual behavior [28]. The motor cortex interacts with the nigrostriatal pathway to fine-tune movement by modulating thalamic activity via the striatum and substantia nigra pars reticulata [29]. This pathway, often interrupted in individuals with SUDs [30], normally acts as a “brake,” ensuring smooth and controlled movement by regulating cortical excitation. Further positive reinforcement in SUDs is provided by the release of the neurotransmitter serotonin from raphe nuclei, which promotes craving and pleasure, driving overall susceptibility [31]. Serotonergic dysregulation, particularly in its projections to the forebrain and PFC, exacerbates impulsivity and mood disturbances, critical factors in addiction and relapse [31].

**Fig. 1:**
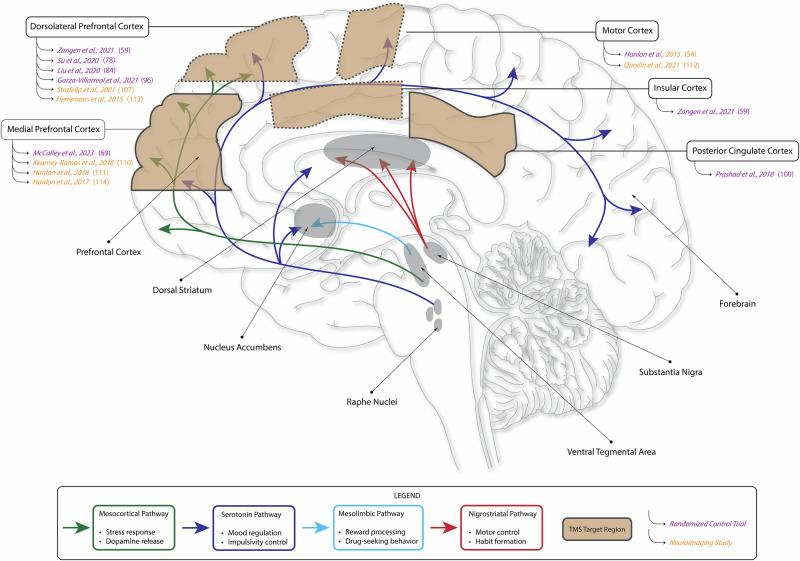
Cortical pathways and brain regions affected in substance use disorders (SUDs) and targeted by transcranial magnetic stimulation (TMS). The mesocortical pathway (green) connects the ventral tegmental area (VTA) to the medial prefrontal cortex (MPFC) and regulates stress responses and dopamine release. The mesolimbic pathway (blue) links the VTA to the nucleus accumbens (NAC) and is essential for reward processing and reinforcement of drug-seeking behavior. The nigrostriatal pathway (red) connects the substantia nigra to the dorsal striatum and is involved in motor control and habit formation. The serotonin pathway (dark purple) originates from the raphe nuclei and projects to the forebrain, influencing mood regulation and impulsivity control. The dorsolateral prefrontal cortex (DLPFC) is involved in executive function and impulse control. The insular cortex plays a role in interoception and craving regulation. The MPFC, cingulate cortex, and motor cortex are involved in decision-making processes, emotional regulation, and motor functions. Together, these circuits and pathways form a complex and vulnerable network that is affected in those with SUDs and thus serves as a therapeutic target for TMS intervention. Select randomized clinical trial and neuroimaging studies discussed in this review are highlighted above in light purple or orange text, respectively, based on the target brain region of the study. *Tan boxes in figure represent brain regions. Boxes outlined in solid lines indicate regions that can be seen in the midsagittal view (MPFC, posterior cingulate cortex) and dotted lines indicate regions that cannot be seen in the midsagittal view (DLPFC, motor cortex, insular cortex). Figure created using Adobe Illustrator*.

Taken together, the dopamine hypothesis of addiction suggests that chronic drug use impairs the standard functioning of several neurotransmitters and brain regions (Fig. 1), namely dopamine and the PFC’s ability to exert control over impulsive behaviors, further driving the cycle of substance use. Functional imaging studies support this hypothesis, showing decreased availability of striatal dopamine receptors and attenuated dopamine release in individuals with SUDs [32]. These changes highlight the profound impact of addiction on brain function and the inherent challenges in restoring baseline dopamine levels and signaling.

By modulating cortical activity, TMS has emerged as a promising therapeutic approach that may potentially restore balance in the brain’s reward circuitry, reducing cravings and improving self-control in individuals with SUDs [33]. This approach leverages our understanding of the neurobiological foundation of addiction to develop targeted, non-invasive treatments that may complement existing therapeutic strategies.

## TMS protocols

TMS is a non-invasive neuromodulation technique that has gained substantial attention for its potential in treating various neuropsychiatric conditions, including SUDs [34, 35]. In the context of addiction, TMS modulates the brain’s underlying reward circuitry within targeted brain regions via variable frequency electrical pulses. For example, stimulating the DLPFC with TMS enhances top-down control over impulsive behaviors to reduce feelings of craving and improves overall decision-making abilities in individuals with SUDs [33]. The electric currents induced by TMS in specific brain regions may also reset the flow of neurotransmitters downstream within deeper brain structures such as the limbic system, though this remains an area of ongoing investigation [36]. Based on the principle of electromagnetic induction, TMS is thought to reduce reward-seeking behavior and diminish the reinforcing effects of illicit substances [37].

Since the 1980s, various protocols have emerged. Repetitive TMS (rTMS) utilizes a coil placed against the scalp delivering magnetic pulses over the cortical region of choice. Stimulation involves the administration of continuous pulses at a certain frequency, or repetitive trains of magnetic pulses, which affect the targeted brain region as well as connected areas [38]. This technique has shown efficacy in treating depression, anxiety, and more recently, SUDs [10, 39]. The ability of rTMS to reach specific brain regions depends on the coil type used. Traditional rTMS employs paddle-shaped figure-8 coils, which generate a highly focal magnetic field that primarily stimulates superficial cortical areas [40]. Computational modeling studies indicate that figure-8 coils produce an electric field with a surface spread as low as 5 cm^2^, however the penetration is superficial, ranging from 1 to 3.4 cm deep [41]. Depending on the frequency and pattern of stimulation, rTMS may either excite or inhibit cortical activity. High-frequency rTMS (at least 5 Hz) is generally excitatory whereas low-frequency TMS (1 Hz or less) is inhibitory [42].

A challenge of standard rTMS therapy and conventional coils is targeting deeper, subcortical brain structures, which has led to the development of deep rTMS (dTMS) [43]. Like rTMS, dTMS involves the application of multiple magnetic pulses over time with excitatory or inhibitory effects depending on the frequency of stimulation but employs specialized coil designs such as the Hesed (H)-coil system [44]. The H-coils have a complex winding pattern encased in a helmet-like apparatus, which conforms to the head’s shape for optimal stimulation stability [45]. This design enhances penetration depth while allowing for the modulation of a broader neural network; however, this comes at the price of reduced focality.

Other advanced coil configurations, such as crown or C-core coils [46], also exist and offer variations in field distribution and depth of penetration, allowing stimulation parameters (e.g., stimulus intensity, number of pulses, frequency) to be tailored to clinical needs. Comprehensive simulations and electric field distributions for 50 TMS coil designs are detailed in the seminal paper by Deng et al. [41], while the structural and functional modeling of the helmet-based H-coil system is described in the patent by Zangen and colleagues. [47].

Differential temporal patterns of stimulation have also been developed including theta burst stimulation (TBS), a refined subtype of rTMS that also primarily utilizes figure-8 coils [48]. TBS delivers three bursts of high-frequency pulses (50 Hz) repeated at theta frequency (5 Hz) and is categorized into two types with opposite effects: intermittent theta burst stimulation (iTBS) and continuous theta burst stimulation (cTBS) [42]. iTBS delivers 600 pulses in total over 190 seconds, enhancing overall neural excitability whereas cTBS delivers 600 pulses in a 40 second train, suppressing neuronal activity [42]. This technique is gaining popularity as an alternative to conventional rTMS protocols due to its shorter administration time and robust effects. A typical rTMS session lasts 30–40 min, while iTBS and cTBS may deliver therapeutic effects in approximately three minutes or less, considerably reducing treatment time [49, 50].

Additional protocols include single-pulse TMS (spTMS) and paired-pulse TMS (ppTMS), which primarily use figure-8 coils to investigate the excitation-inhibition balance in specified brain regions. spTMS involves the delivery of individual magnetic pulses to measure motor evoked potentials and the general excitability of the corticospinal tract [51, 52]. ppTMS utilizes two successive magnetic pulses with a variable interval to gain insights into synaptic interactions and cortical connectivity [53]. Investigators utilize these techniques to map neurophysiological changes and better understand neural integrity disruptions. For instance, ppTMS has been instrumental in revealing that chronic cocaine users exhibit higher motor thresholds and cortical facilitation, reflecting altered motor cortex excitability [54]. These disruptions help explain the hyperkinetic symptoms of individuals with cocaine use disorders as well as their disrupted synaptic function and overall neuroplasticity [54].

TMS, regardless of the specific protocol used, is a non-invasive and well-tolerated procedure with minimal discomfort for most patients. The most reported side effects, such as mild scalp discomfort or transient headaches, are typically minor and resolve quickly without intervention. Although seizures have been reported in rare cases, the overall risk remains extremely low, particularly when safety guidelines regarding stimulation intensity and frequency are followed [55, 56]. Due to its favorable safety profile and non-invasive nature, TMS is widely considered a well-tolerated neuromodulatory intervention, making it a viable therapeutic option for a broad range of patients.

**Fig. 2:**
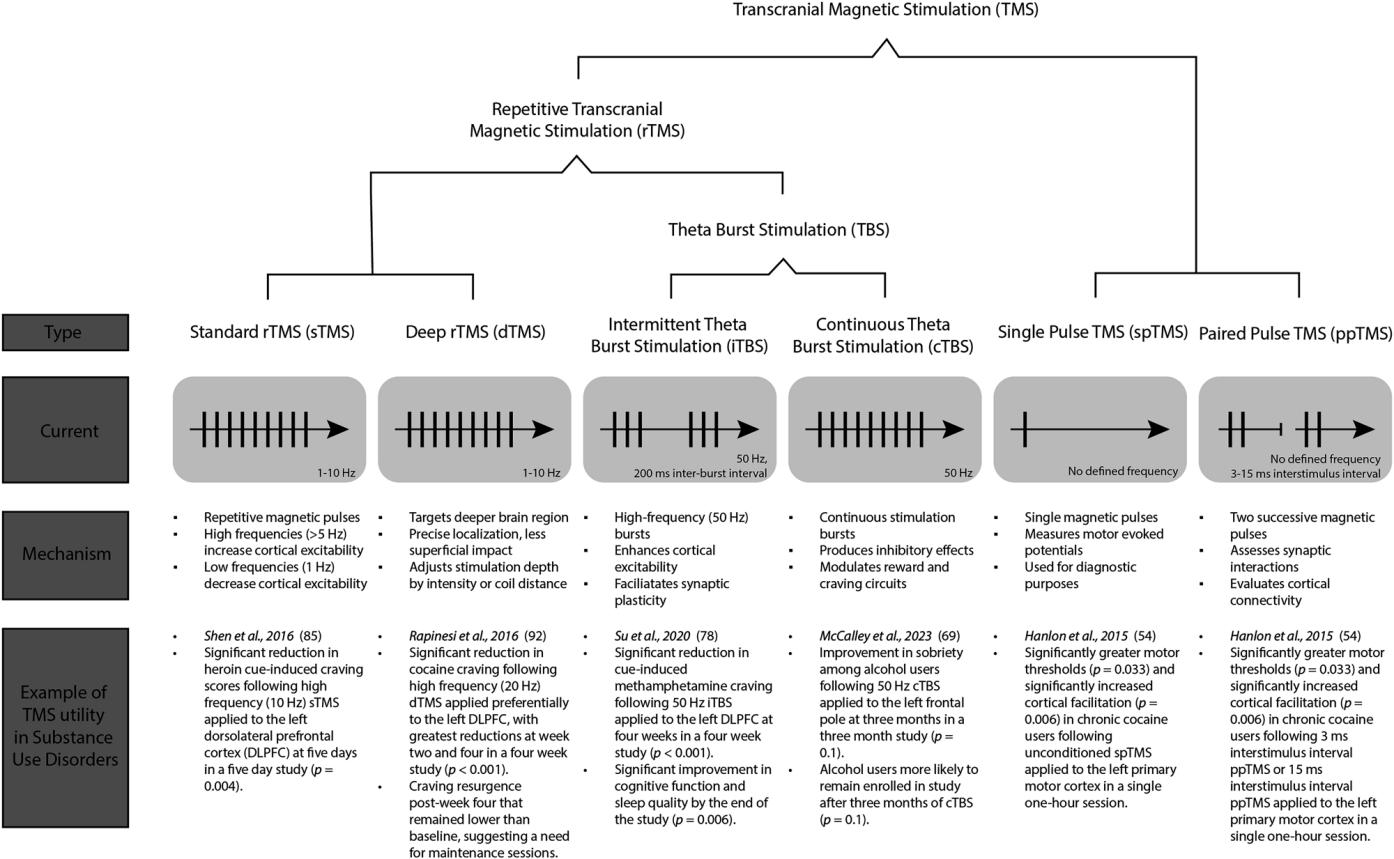
Different types of transcranial magnetic stimulation (TMS) protocols in substance use disorders (SUDs). Various TMS protocols have been used to treat SUDs including standard repetitive TMS (rTMS), deep rTMS (dTMS), intermittent theta burst stimulation (iTBS), continuous theta burst stimulation (cTBS), single pulse TMS (spTMS), and paired pulse TMS (ppTMS). Examples of randomized clinical trials (RCTs) that utilized each respective TMS protocol for SUD treatment are listed. Each protocol is characterized by different patterns of magnetic pulses, frequencies, and mechanisms of action. Standard rTMS utilizes repetitive magnetic pulses at constant frequencies (1–10 Hz), increasing or decreasing cortical excitability based on the frequency used. dTMS employs specialized coils for targeting deeper brain structures, offering localization with minimal impact on the cortex. iTBS delivers high-frequency bursts (50 Hz, with inter-burst intervals) to enhance cortical excitability and facilitate synaptic plasticity. cTBS applies continuous bursts (50 Hz) that produce inhibitory effects and modulate reward-related neural circuits. spTMS and ppTMS are primarily diagnostic, with spTMS delivering single pulses to measure motor evoked potentials and ppTMS using paired pulses (with 3–15 ms interstimulus intervals) to assess synaptic interactions and cortical connectivity.

In summary, TMS is a versatile neuromodulation technique that influences cortical excitability and neural circuits implicated in neuropsychiatric illnesses and addiction-related behaviors. Over the past 40 years, advancements in stimulation protocols, coil designs, and temporal patterns have significantly expanded its clinical applications. The introduction of dTMS, TBS, and targeted spTMS and ppTMS paradigms (Fig. 2) has improved stimulation depth, efficiency, precision, and knowledge of underlying physiological changes, making TMS an increasingly adaptable tool for treating SUDs. While further research is needed to refine optimal protocols and patient-specific treatment approaches, numerous clinical and neuroimaging studies support the efficacy and safety of TMS across diverse population samples.

## Clinical studies assessing TMS in the treatment of SUDs

Clinical studies investigating the efficacy of TMS in SUD treatment have yielded promising results. RCTs have demonstrated the potential of TMS to reduce cravings and substance use as well as decrease relapses across various addiction types including tobacco, alcohol, methamphetamines, opioids, cocaine, and cannabis [57, 58]. Although several RCTs are discussed per substance, a representative RCT, defined as one that aligns with the broader findings, is highlighted for each SUD in Table 1.

**Table 1:** Prominent recent randomized clinical trial studies showing efficacy of transcranial magnetic stimulation (TMS) in substance use disorders (SUDs).

Study	SUD	TMS Type	Brain Region	Coil Placement	Main Findings
*Zangen* et al. [59]	Tobacco	Repetitive transcranial magnetic stimulation (rTMS)	Lateral prefrontal cortex and insular cortex	Coil placed to localize the optimal helmet position for activation of the right abductor pollicis brevis muscle, then aligned symmetrically and moved 6 cm anteriorly.	• Quit rate was significantly greater among tobacco users in the active 10 Hz rTMS group (63%) compared to the sham group (50%) at week 18 (*p* = 0.003). • Significantly greater reduction in cigarette consumption and craving was seen in the active compared to sham group by week 2 of treatment (*p* = 0.022).
*McCalley* et al. [69]	Alcohol	Continuous theta burst stimulation (cTBS)	Medial prefrontal cortex (MPFC)	Coil placed over FP1, a standard EEG site correlating to the left frontal pole.	• Participants receiving cTBS were 3.09 times more likely to remain sober after three months and 2.71 times more likely to remain enrolled in the study after three months (*p* = 0.10). • Significant reduction in MPFC-striatum and MPFC-insula connectivity—dysfunctional circuits associated with habit formation and emotion, respectively—two-three months post-treatment (*p* < 0.05).
*Su* et al. [78]	Methamphetamine	Intermittent theta burst stimulation (iTBS)	Left dorsolateral prefrontal cortex (DLPFC)	Coil placed over F3, a standard EEG site correlating to the left DLPFC.	• Significant reduction of cue-induced methamphetamine craving in iTBS (50 Hz bursts) compared to sham group after four weeks (*p* < 0.001) • Significant improvement of cognitive function (*p* < 0.001) and sleep quality (*p* = 0.006).
*Liu* et al. [84]	Opioid (heroin)	rTMS	Left DLPFC	Coil placed over F3 using the 10–20 EEG system.	• 1 Hz and 10 Hz rTMS significantly reduced cue-induced heroin craving in users compared to controls at treatment day 30, 60, and 90 (*p* < 0.0001). • Effects lasted up to 60 days post-treatment.
*Garza-Villarreal* et al. [96]	Cocaine	rTMS	Left DLPFC	Coil placed 5 cm along and 2 cm anterior to the interaural line, at a 45° angle with respect to the interhemispheric fissure; some participants received the F3 method.	• Significant reduction in cocaine craving (*p* = 0.013) and impulsivity (*p* = 0.011) among cocaine users in the 5 Hz rTMS compared to sham group after two weeks. • Functional connectivity between the DLPFC and ventromedial PFC increased, particularly in the active group, but was not significant over three months (*p* > 0.05).
*Prashad* et al., 2018 [100]	Cannabis	rTMS	Posterior cingulate cortex (PCC)	Coil placed 4 cm behind the motor strip using a double-cone coil (DCC).	• Increased responses to self-relevant stimuli were significantly reduced among cannabis users administered 10 Hz rTMS compared to the sham-stimulated group (*p* < 0.05). • No significant effect of rTMS on cannabis-stimuli (*p* > 0.05).

Studies spanning multiple TMS modalities including single-pulse (spTMS), paired-pulse (ppTMS), repetitive (rTMS), continuous theta burst stimulation (cTBS), and intermittent theta burst stimulation (iTBS), and multiple brain regions implicated in SUDs including various sub-regions of the prefrontal cortex, motor cortex, insular cortex, and cingulate cortex. These regions are distinctly shown in Fig. 1. Coil placement strategies and key findings from each study are outlined, highlighting reductions in cravings, changes in neural connectivity, and improvements in cognitive function.

In 2020, Zangen and colleagues applied deep TMS to the lateral PFC and insular cortex utilizing the H-4 coil in patients with tobacco use disorder and found significant reductions (*p* < 0.05) in craving and consumption compared to the sham stimulated cohort [59]. This study is the only large multicenter RCT to date in the addiction neuromodulation field and has led to FDA clearance of use of the H-4 coil for smoking cessation [9]. Several clinical trials from the years 2003–2018 demonstrated efficacy of rTMS for tobacco use disorder reporting reductions in tobacco craving and consumption following active versus sham high frequency rTMS stimulation administered with various coil types to regions of the PFC and insular cortex [60–68]. However, the sample sizes in these studies ranged from *n* = 14 to *n* = 77 and warrant studies with larger cohorts, like the one by Zangen and colleagues [59], to determine standardized, precise, safe, and efficacious protocols necessary for FDA approval of TMS therapy in tobacco use disorder.

A recent double-blind, sham controlled RCT investigated the effects of cTBS on the effect of alcohol use disorder and reported that at least 10 sessions targeting a specific electrode site (labeled FP1) of the MPFC region were necessary to achieve significant reductions in cravings (as measured by the obsessive-compulsive drinking scale) and significantly decreased brain reactivity to alcohol cues (as measured by the alcohol cue fMRI task) among patients with alcohol use disorder compared to a sham-stimulated group (*p* < 0.05) [69]. Although cTBS is generally inhibitory, this finding aligns with RCTs using different TMS protocols which consistently demonstrate that at least 10 sessions of high frequency (10–20 Hz) stimulation applied to the MPFC, DLPFC, or insular cortex are necessary to produce significant reductions (*p* < 0.05) in alcohol craving and / or consumption compared to sham stimulation [70–73]. The variability in stimulation approaches reflects the complex pathophysiology of alcohol use disorder, where inhibitory TMS may be used to reduce excessive cue-reactivity when the MPFC is hyperactive, as in the former study, whereas excitatory TMS may be used to restore impaired cognitive control or to enhance top-down regulation over cravings when certain brain regions are hyperactive, like in the latter studies. Both approaches address distinct but complementary challenges.

Like alcohol use disorder, numerous studies exhibit considerable improvements in cravings and consumption among patients with methamphetamine use disorder with the application of multiple (at least five), variable frequency (1–10 Hz) rTMS sessions targeting the DLPFC or PFC in general [74–80]. This holds true across studies with standard rTMS application as well as iTBS and cTBS therapies. These findings support the use of MPFC, DLPFC, and insula-targeted TMS therapy to decrease use, cravings, and compulsions in SUDs, but warrant studies with larger cohorts to standardize treatment for alcohol and methamphetamine addiction.

In opioid use disorder, RCTs have shown promise in reducing cravings specifically with left DLPFC-targeted TMS treatment. This effect may be attributed to the role of this brain region in executive functioning, decision making (specifically craving reduction), and overall dopaminergic modulation [81, 82]. One study observed reduced opioid cravings in conjunction with improvements in depressive symptoms after multiple high-frequency rTMS sessions, although this was administered with concurrent occupational therapy [83]. Two studies investigated the treatment effects of isolated high-frequency rTMS sessions in patients with opioid use disorder (heroin) and still demonstrated significantly reduced (*p* < 0.05) cravings and cue-induced cravings compared to sham stimulated individuals [84, 85]. In fact, multiple low (1 Hz) and high (10 Hz) frequency rTMS sessions produced a significant reduction (*p* < 0.05) in cue-induced opioid cravings compared to no treatment, suggesting the efficacy of consistent left DLPFC-mediated TMS therapy in this population. Larger clinical trials showing similar positive effects with the use of specified coils in this brain region, as well as other implicated brain regions, are necessary to further understand efficacious TMS protocols in opioid use disorder.

RCTs exploring the effects of TMS in cocaine use disorder have consistently shown reductions in cravings and cocaine use. In the early 2000s, two independent studies reported a significant reduction (*p* < 0.05) in cocaine craving after either a single session of high frequency (10 Hz) rTMS stimulation applied to the right DLPFC [86] or multiple (*n* = 10) sessions of high frequency (15 Hz) rTMS stimulation applied to the left DLPFC [87]. Neither of these studies specified the coil type utilized. From 2016 to 2020, several clinical trials reported that multiple sessions (at least eight) of high frequency (at least 10 Hz) deep rTMS or iTBS applied to the DLPFC, MPFC, or overall PFC over a period of at least five consecutive days reduced cocaine craving, intake, and/or elongated the latency to first relapse in the TMS-treated group compared to the sham stimulated group [88–95]. In addition, several of the most recent RCTs (since 2021) report similar findings. In a double-blind RCT, 44 patients with cocaine use disorder were split into treatment and control groups, with the treatment group receiving two daily sessions of 5-Hz rTMS on the left DLPFC for two weeks. The study concluded that 5-Hz rTMS reduced impulsivity and subjective craving of cocaine in the participants [96]. A randomized, double-blinded study utilized 15-Hz rTMS on the left DLPFC in half of their cocaine use disorder patients for three weeks. Eight weeks after treatment, the study reported a significant decrease (*p* < 0.05) in self-reported cocaine use in this group compared to a sham stimulated group [11]. In line with these investigations, two systematic reviews on the effects of TMS on cocaine addiction found a significant decrease (*p* < 0.05) in impulsivity and cocaine craving [42, 97]. In all these reports, TMS was efficacious and well-tolerated among cocaine use disorder patients, albeit with little information regarding the coil type used to observe results.

Less focused efforts have been made towards investigating the utility of TMS in cannabis use disorder relative to other SUDs. This may be due to several reasons, one being that while cannabis use is common, the proportion of users who develop a disorder is relatively small compared to substances like nicotine or alcohol [98]. In addition, research funding is often directed towards substances with higher public health impact and associated costs [99]. To date, two randomized, sham-control trials have examined the use of rTMS in cannabis users utilizing a single high frequency (10 Hz) session [100, 101]. One study applied rTMS to the left DLPFC [101] while the other study targeted the posterior cingulate cortex and precuneus [100]. The latter study additionally tested the effect of low frequency (1 Hz) rTMS; however, these studies did not find statistically significant differences (*p* > 0.05) in cannabis use or craving between the rTMS and sham groups. Another study applied multiple (*n* = 20) sessions of high frequency (20 Hz) rTMS to the right and left DLPFC in participants with comorbid cocaine use disorder and schizophrenia, reporting no statistically significant difference (*p* > 0.05) in cannabis use between active and sham stimulation groups [102, 103]. Further research into cannabis in general as well as larger RCTs are warranted to investigate the efficacy of TMS for cannabis users, but preliminary studies have not reached statistical significance with predominantly DLPFC-targeted TMS therapy.

The RCTs reviewed above demonstrate the potential of TMS in treating SUDs; however, several challenges persist that limit FDA approval and systematic TMS utility. Many of these studies have small sample sizes and exhibit methodological limitations such as lack of control groups. The lack of standardized TMS protocols across studies leads to variability in treatment parameters such as target brain regions, coil types, stimulation frequencies, and the number of sessions. While methodological heterogeneity poses challenges for direct comparisons among studies, the accumulated data from existing RCTs, particularly those with larger sample sizes (*n* > 20) and varied stimulation parameters, provide a foundation for systematic evaluation. Leveraging meta-analytic approaches, such as random-effects models and dose-response meta-regression, may help quantify sources of heterogeneity and identify optimal stimulation parameters for specific SUDs. In addition, clinical trials may minimize this inherent heterogeneity across studies by implementing standardized procedures and conducting sufficiently powered trials with control groups. Variations in individual trials could be limited to parameters such as the target brain region, method of accessing it, coil type, frequency, number of pulses, and number of TMS sessions. Comparisons should be made between studies of similar sample sizes with similar controlled variables. Such efforts will be essential to optimize the efficacy and clinical utility of TMS as a neuromodulatory treatment for SUDs.

## The utility of neuroimaging to inform TMS therapy for SUDs

Neuroimaging modalities, such as positron emission tomography (PET) and functional magnetic resonance imaging (fMRI), offer invaluable tools for monitoring brain activity during TMS treatment [104]. These techniques enable the detection of real-time structural changes and connectivity patterns, which may serve as biomarkers or predictors of treatment response, thereby refining and personalizing TMS protocols. Notably, neuroimaging has demonstrated that the effects of TMS extend beyond the initial stimulation sites (i.e., target brain regions), influencing interconnected neural networks and broader brain circuits [105, 106]. Despite these advancements, a challenge lies in consistently translating neuroimaging insights into standardized, evidence-based interventions for patients with SUDs. In this section, we review studies that successfully integrated neuroimaging with TMS (Table 2), highlighting its potential as a critical component in the development of effective, individualized treatments for SUDs. Beyond demonstrating treatment effects below, we discuss how these neuroimaging findings may inform TMS target selection.

**Table 2:** Key neuroimaging studies highlighting the role of various imaging techniques in guiding transcranial magnetic stimulation (TMS).

Study	Imaging Modality	Population	TMS Target	Coil Placement	Key Findings	Relevance
*[11 C]raclopride PET study (Strafella* et al., *2001*)) [106]	PET	Healthy individuals	Left mid-dorsolateral prefrontal cortex (MDL-PFC) and left occipital cortex	Circular coil (9 cm diameter), anterior tip closest to the cortical site, held in place by a mechanical arm	7.3% reduction in [11 C]raclopride binding in ipsilateral caudate nucleus, indicating increased dopamine release.	Highlights PET’s utility in tracking dopamine release for TMS efficacy in SUDs.
*Structural brain integrity study (Kearny-Ramos* et al., *2018*) [109]	fMRI, DTI, VBM	Cocaine-dependent individuals	Left ventromedial prefrontal cortex (VMPFC)	Figure-of-eight coil, positioned at FP1 location using EEG 10–20 system, mounted in MR head coil with a custom holder	TMS-evoked BOLD responses in striatal regions (e.g., caudate, putamen) correlated with 15% higher fractional anisotropy in white matter tracts and 12% greater gray matter volume in the VMPFC.	Establishes white and gray matter integrity as biomarkers for optimizing TMS efficacy.
*Sensorimotor excitability study (Hanlon* et al., *2020*) [53]	fMRI	Chronic cocaine users	Left primary motor cortex	Magstim D70 Alpha coil, held tangential to the scalp with handle at 45° from midline, secured using Brainsight coil and head support system	Resting motor thresholds were 10.8% higher in cocaine users, while intracortical facilitation was elevated by 15%, linked to increased BOLD signals in motor cortices.	Demonstrates TMS-fMRI integration to refine protocols for disrupted cortical facilitation in SUDs.
*Drug cue-reactivity study (Hanlon* et al., *2018*) [110]	fMRI	Individuals with cocaine, alcohol, or nicotine dependence	EEG 10-10 system coordinates based on drug cue activation hotspots: usually medial prefrontal cortex (MPFC)	Determined by Euclidean distances to nearest EEG 10-10 coordinates, varying by 2–5 cm depending on coil penetration characteristics	Drug-related cues increased MPFC activation in 40% of participants; MPFC ‘hotspots’ were within two–five cm of scalp landmarks for 49% of individuals.	Identifies individualized TMS targets for SUDs using cue-elicited brain activity.
*Structural MRI in alcohol use disorder (Quoilin* et al., *2021*) [111]	Structural MRI	Detoxified alcohol-dependent patients	Right primary motor cortex (M1)	Figure-of-eight coil (70 mm wing diameter), tangential to scalp with handle at 45° angle from midline, perpendicular to central sulcus	Cortical thickness reductions of 20% in MPFC and 15% in SMA correlated with weaker preparatory suppression and poorer behavioral inhibition, respectively.	Links structural deficits to motor and cognitive dysfunctions, informing TMS biomarker selection.
*HF-rTMS in alcohol-dependent patients (Herremans* et al., *2015*) [112]	fMRI	Detoxified alcohol-dependent patients	Right dorsolateral prefrontal cortex (DLPFC)	Figure-of-eight coil (70 mm), tangential to skull, positioned with MRI-guided non-stereotactic method, perpendicular projection on scalp	15 HF-rTMS sessions reduced general craving scores by 25% but showed no significant change (*p* > 0.05) in cue-induced craving; left inferior parietal lobule activity decreased by 18%.	Reveals fMRI’s role in tracking indirect TMS effects on attentional and reward networks.
*cTBS effects in cocaine and alcohol dependence (Hanlon* et al., *2017*) [113]	BOLD fMRI	Cocaine and alcohol-dependent individuals	Left frontal pole (FP1)	Magstim double 70 mm coil, positioned at FP1 using EEG 10–20 system, mounted in MR head coil with custom holder	cTBS decreased evoked BOLD responses by 22% in caudate, 18% in nucleus accumbens, and 20% in orbitofrontal cortex in cocaine users; alcohol-dependent individuals showed 25% reduction in anterior insula activity.	Supports fMRI in guiding TMS for modulating salience circuits in SUDs.

Studies showcasing the integration of neuroimaging with TMS protocols to enhance therapeutic outcomes. The table outlines the imaging modality used, the population studied, TMS stimulation sites, coil placement methods, key findings, and the relevance of each study, emphasizing evidence for neuroimaging’s role in optimizing TMS targets, tracking treatment effects, and personalizing interventions for SUDs.

*PET imaging of dopamine release in response to TMS*: An investigation utilizing [^11^C]raclopride, a dopamine D_2_ / D_3_ receptor antagonist used in PET imaging to measure extracellular dopamine levels, measured changes in extracellular dopamine concentration following rTMS of the left DLPFC in healthy individuals [107]. The study reported a significant (*p* < 0.05) 7.3% reduction in [^11^C]raclopride binding potential in the ipsilateral caudate nucleus compared to the occipital cortex, showing increased dopamine release in response to rTMS. No significant changes (*p* > 0.05) were observed in the putamen, nucleus accumbens, or contralateral caudate nucleus. These results suggest that rTMS modulates dopamine release primarily through direct corticostriatal projections originating in the DLPFC. The findings implicate the potential therapeutic benefits of rTMS in disorders characterized by dopamine dysregulation, such as SUDs, and demonstrate the utility of [^11^C]raclopride as a biomarker for tracking TMS-induced changes in dopamine activity.

*Integrated neuroimaging to assess brain tissue integrity in TMS*: The first study to examine how structural brain integrity influences TMS efficacy in modulating subcortical activity utilized a multimodal approach combining fMRI, diffusion tensor imaging (an MRI-based technique that maps white matter integrity by measuring the diffusion of water molecules along axonal pathways, providing insight into structural connectivity [108]), and voxel-based morphometry (a neuroimaging method that assesses regional gray matter volume and density, allowing for comparisons of structural differences [109]) in cocaine-dependent individuals [110]. Single pulses of TMS applied to the left ventromedial prefrontal cortex (VMPFC) evoked significant (*p* < 0.05) blood oxygen level-dependent (BOLD) responses in striatal regions such as the caudate and putamen, as well as in the salience network, including the anterior cingulate cortex (ACC) and insula. Notably, the magnitude of TMS-evoked striatal activity was positively correlated with fractional anisotropy values, a measure of the diffusion tensor imaging technique, along white matter tracts connecting the VMPFC to the striatum, indicating stronger, more organized connections. Similarly, gray matter volume at the stimulation site (VMPFC) and the left ACC was significantly associated with greater BOLD responses in these regions (*p* < 0.05). These findings highlight the importance of white and gray matter integrity as biomarkers for optimizing TMS efficacy in populations with structural brain deficits. By highlighting the critical role of structural neuroimaging in guiding TMS application, this study establishes a framework for patient-specific TMS protocols aimed at mobilizing subcortical targets in SUD treatment.

*fMRI-guided TMS to decipher neurophysiological changes*: In 2015, Hanlon et al. utilized TMS and fMRI to investigate sensorimotor excitability in chronic cocaine users during a finger-tapping task [54]. The study revealed two key findings: (I) chronic cocaine users exhibited significantly higher (*p* < 0.05) resting motor thresholds (44 ± 6.4%) compared to controls (39.7 ± 5.4%), indicating reduced cortical excitability; and (II) heightened intracortical facilitation in cocaine users was positively correlated with elevated BOLD signals in the left and right primary motor cortices during the task. Notably, these differences were specific to excitatory mechanisms, as measures of inhibitory tone (e.g., cortical silent period) showed no significant (*p* > 0.05) group differences. The integration of TMS and fMRI allowed the identification of disrupted cortical facilitation as a surrogate for abnormal task-evoked motor activity, demonstrating the importance of multimodal imaging in refining TMS protocols and aligning them with specific neural dysfunctions in cocaine use disorder.

*fMRI to investigate brain activity during TMS*: A few years later, Hanlon et al. again conducted a large study using fMRI during a standardized drug-cue reactivity task to investigate cortical “hot spots” of activity in 156 individuals dependent on cocaine, alcohol, or nicotine [111]. Drug-related cues elicited significantly increased BOLD signals in three key regions: the MPFC (Brodmann Area [BA] 10), left inferior frontal gyrus/insula (BA 44), and right premotor cortex (BA 8) (*p* < 0.05). Using k-means clustering, the study identified the MPFC as the most consistent activation site across all substance use groups, with 40% of participants exhibiting peak activity in this region. To evaluate the accessibility of these hot spots for TMS, the researchers calculated their proximity to scalp landmarks and found that the MPFC was within a 2–5 cm range for 49% of participants. These findings reveal the critical role of neuroimaging in identifying individualized TMS targets based on cue-elicited brain activity, facilitating precise protocol optimization for individuals with multiple SUDs where connectivity disruptions vary widely.

*Structural MRI to link structural-behavioral patterns*: Supporting these findings, structural MRI has advanced the understanding of inhibitory control deficits in alcohol use disorder by identifying cortical morphology changes linked to motor and cognitive impairments. A study of 45 detoxified patients with alcohol use disorder used structural MRI with TMS to quantify preparatory suppression, a motor excitability regulation measure [112]. Results revealed that reduced cortical thickness in the MPFC, encompassing the ACC and superior frontal gyrus, correlated with weaker preparatory suppression, while thinning in the supplementary motor area (SMA) and pre-SMA was associated with poorer behavioral inhibition. These results show the utility of neuroimaging in linking structural brain changes to deficits in motor and cognitive functions in patients with alcohol use disorder and highlight its potential to identify biomarkers for tailoring TMS interventions.

*fMRI to detect TMS-induced brain changes*: In recently detoxified alcohol-dependent patients, neuroimaging revealed how accelerated high-frequency rTMS (HF-rTMS) impacts brain activity. A study employing HF-rTMS targeting the right DLPFC alongside fMRI utilized block and event-related alcohol cue-reactivity paradigms to examine changes in craving and associated neurocircuitry [113]. While 15 HF-rTMS sessions over four days significantly reduced (*p* < 0.05) general craving as assessed by scales like the Alcohol Urge Questionnaire and Obsessive-Compulsive Drinking Scale, cue-induced craving scores remained unchanged. Neuroimaging revealed no significant alterations (*p* > 0.05) in the core craving neurocircuit following stimulation but identified reduced activation in the left inferior parietal lobule—a region linked to salience attribution and sensory representation—during event-related paradigms. In this case, fMRI successfully discerned HF-rTMS’s indirect modulation of attentional and reward networks, aiding in the refinement of TMS protocols for individualized therapeutic interventions.

*BOLD fMRI to investigate TMS-induced brain changes*: Neuroimaging with BOLD fMRI has been critical in detailing the effects of cTBS on brain activity in cocaine- and alcohol-dependent individuals. In another study by Hanlon et al., six trials of cTBS delivered to the left frontal pole significantly decreased evoked BOLD responses in regions central to the salience and reward networks (*p* < 0.05) [114]. Cocaine users exhibited reduced activity in the caudate, NAC, ACC, and orbitofrontal cortex, while alcohol-dependent individuals showed decreased activity in the orbitofrontal cortex, anterior insula, and lateral sensorimotor cortex. These findings again show the utility of BOLD fMRI in identifying neural regions influenced by cTBS and guiding its application in modulating overactive salience circuits associated with SUDs. This multimodal approach reveals the neural mechanisms underlying TMS-induced changes in SUDs, informing the advancement of targeted interventions.

*Neuroimaging-guided TMS brain region selection*: As demonstrated, neuroimaging modalities offer invaluable tools for monitoring brain activity during TMS treatment. They are also instrumental in identifying optimal cortical targets for intervention. PET imaging of dopamine release confirmed that rTMS applied to the DLPFC modulates corticostriatal projections, validating this key intervention site [107]. Diffusion tensor imaging identified that stronger connectivity between the MPFC and striatal regions predicts greater TMS-induced reductions in craving, reinforcing the importance of targeting circuits with intact structural pathways for optimal intervention [110]. fMRI drug-cue reactivity studies validate greater activation of the MPFC and ACC across SUDs. Thus, neuroimaging not only tracks TMS-induced changes but also validates optimal intervention targets, allowing for precision-medicine approaches to TMS in SUD treatment [111, 115].

These studies demonstrate the potential of TMS as an extremely versatile and effective therapeutic intervention for SUDs when combined with neuroimaging techniques. By providing critical insights into structural integrity, functional connectivity, and activity patterns across key brain regions, neuroimaging allows for the identification of biomarkers and personalized targets, optimizing TMS protocols for maximum efficacy. This integration enhances our understanding of the neural mechanisms underlying SUDs and also paves the way for evidence-based, patient-specific treatments. Future research should explore how the integration of multimodal neuroimaging approaches with personalized TMS protocols may refine TMS applications and expand its therapeutic reach in addressing the complex neurocircuitry underlying SUDs.

## Challenges & future directions

While converging evidence highlights the therapeutic potential of TMS for SUDs, further research is needed to standardize optimal parameters, biomarkers, and neuroimaging measurements. Current challenges include variability in stimulation parameters, lack of standardized protocols, and a limited understanding of individual differences in treatment response [116]. Variability arises from the use of different coils (i.e., H-coil vs. figure-of-8), targeting different brain regions (i.e., insula vs. DLPFC), as well as varying intensities, frequencies, and durations of stimulation sessions [117].

Identifying the six “known unknowns” in TMS research—cortical target selection, subcortical circuit engagement, optimizing rTMS sequences, rTMS as an adjuvant to existing interventions, manipulating brain state, and selection of outcome measures—is crucial [116]. Structuring translational research to address these factors may help standardize protocols in these domains. For example, “manipulating brain state” refers to the influence of baseline neural activity, cognitive engagement and physiological conditions (e.g., withdrawal states or medication use) on TMS responsiveness. Preliminary evidence suggests that TMS stimulation effects vary depending on whether the subject is in a resting vs. task-engaged state, highlighting the importance of further studies on state-dependent neuromodulation strategies in SUD treatment [118]. In addition, individual differences in neurobiology and clinical presentation such as polysubstance use disorder, comorbid psychiatric disorders, use of prescribed medications, as well as anatomical differences, like skull thickness and cerebrospinal fluid distribution complicate treatment standardization and must be considered to appreciate TMS outcomes [57, 116, 119, 120].

Standardized outcome measures are essential for associating results across comparative studies and establishing evidence-based guidelines for optimal SUD treatment. Current studies rely on subjective measures like self-reported cravings, inconsistent use of objective measures like drug tests, and overlook long-term effects such as relapse rates [89, 117]. Combining TMS with neuroimaging studies, however, may lead to biomarkers for SUDs especially when used with pharmacologic intervention, providing reliable insights into brain structure and connectivity, helping to personalize therapy, predict treatment responses, and pave the future for TMS as a standardized, mainstream treatment option for SUDs [119, 120]. Furthermore, future meta-analyses incorporating multilevel modeling and stratified subgroup analyses could further refine parameter-efficacy relationships, providing a structured framework for optimizing TMS protocols in SUD treatment.

## Conclusion

Over the past 20 years, the TMS field has expanded significantly, evolving from showing promising research findings to having several FDA-approved medical devices used to treat a wide range of neuropsychiatric disorders such as major depressive disorder and obsessive-compulsive disorder. Given the recent FDA approval for H-4 coil use in smoking cessation, TMS holds promise as a treatment modality for SUDs. Growing evidence demonstrates the anti-craving, consumption-reducing, and impulsivity-reducing effects of this therapy through modulation of the neurocircuitry underlying addiction such as the mesolimbic, mesocortical, and nigrostriatal pathways. Inconsistencies in the methodology of current TMS studies as well as individual anatomical, physiological, and behavioral differences limit the ability to compare results across studies; thus, future research should focus on refining TMS protocols and integrating neuroimaging modalities to further reveal the neural mechanisms of action and identify biomarkers of TMS treatment response for SUDs. Overall, TMS represents a promising avenue for advancing treatment for addiction, offering new hope for individuals struggling with SUDs.

## Data Availability

All data is available within the Review Article.

## Reproducibility Statement

The authors confirm that all content in the manuscript is original and has not been submitted elsewhere for publication consideration.
